# Early and Emergency Department Management of Infraclavicular Brachial Plexus Injuries Secondary to Shoulder Trauma

**DOI:** 10.7759/cureus.110594

**Published:** 2026-06-10

**Authors:** Praveen Baskaran, Abigail S Bower, Ashley I Simpson

**Affiliations:** 1 Trauma and Orthopaedics, Barnsley Hospital NHS Foundation Trust, South Yorkshire, GBR; 2 Department of Emergency Medicine, Doncaster Royal Infirmary, South Yorkshire, GBR; 3 Peripheral Nerve Injury, Royal National Orthopaedic Hospital, London, GBR

**Keywords:** brachial plexus, emergency medicine, infraclavicular, medicolegal documentation, neurological examination, peripheral nerve injury, proximal humerus fracture, shoulder dislocation

## Abstract

Infraclavicular brachial plexus injury is an uncommon but potentially disabling complication of shoulder trauma, particularly glenohumeral dislocation and proximal humerus fracture or fracture-dislocation. Reported frequencies of neurological deficit vary widely because of heterogeneous definitions and ascertainment methods. Systematic reviews consistently identify the axillary nerve as the most commonly affected nerve following dislocation; however, multi-nerve deficits indicating cord-level involvement are clinically significant because recovery may be delayed or incomplete.

Emergency care priorities for suspected infraclavicular brachial plexus injury centre on early, repeated, and well-documented neurological examination mapped to named cords and terminal branches, alongside prompt, gentle reduction of dislocations when a nerve deficit is present. Analgesia that facilitates examination without obscuring baseline deficits and imaging should be tailored to the fracture pattern and assessment of associated vascular red flags. Time-sensitive referral to orthopaedic and specialist peripheral nerve services is essential when red flags are identified. Concurrent vascular injury is a critical red flag that must be recognised early and escalated because it may compound nerve injury through compression or ischaemia and is itself limb-threatening. UK BOASt (British Orthopaedic Association Standards for Trauma) standards emphasise documentation of nerve function at the earliest opportunity and following interventions, alongside efficient referral pathways.

This article is a narrative review with a pragmatic emergency department pathway proposal. It synthesises systematic reviews, large observational studies, and UK national standards. The proposed pathway is an expert-opinion synthesis that incorporates checklist-based documentation, staged imaging, and clear thresholds for escalation and specialist referral. It is intended for local adaptation and audit and has not been prospectively validated. The aim is to reduce missed injuries, optimise time-sensitive reconstructive opportunities, and mitigate medicolegal risk.

## Introduction and background

Shoulder dislocation is a common injury worldwide and contributes substantially to emergency department presentations and disability burden [1,2]. Analyses derived from Global Burden of Disease data estimate a crude incidence of approximately 75 per 100,000 persons in 2019, with a bimodal age distribution, including peaks in young men and older women [1,2]. Proximal humerus fractures represent a related pattern of shoulder trauma, particularly in older adults, and may similarly be complicated by neurovascular injury; systematic review data report complication rates ranging from 0.09% to 5%, with vascular injury potentially occurring in the absence of classical clinical signs [3,4]. Both injury patterns may therefore be complicated by neurological injury, and infraclavicular brachial plexus injury (IBPI) represents a specific and underappreciated consequence encountered in emergency practice [5,6].

Neurological deficits following glenohumeral dislocation are variably reported, reflecting differences in examination practices and the use of electrodiagnostic testing [5,6]. These injuries exist along a spectrum, from isolated terminal nerve involvement, most commonly axillary neuropraxia, to true IBPI, a distinct and more serious entity involving cord-level elements of the plexus [5,6].

A brief anatomical overview clarifies this distinction. The brachial plexus is divided by the clavicle: the roots and trunks lie superiorly, whilst the infraclavicular plexus comprises the lateral, posterior, and medial cords, together with their terminal branches. Isolated mononeuropathies generally carry a favourable prognosis. In contrast, IBPI often produces a multi-nerve deficit and may result in substantial long-term functional impairment with incomplete neurological recovery [6].

IBPI remains under-recognised in emergency practice, partly because neurological deficits following shoulder dislocation are frequently attributed to isolated axillary neuropraxia [5,6]. Failure to distinguish between these injuries carries important prognostic implications, as early recognition, appropriate investigation, and timely referral may influence functional outcomes [5]. Despite the potentially significant consequences of delayed diagnosis, practical guidance focused on the early recognition and initial management of IBPI in the emergency setting remains limited. Emergency clinicians therefore require a practical framework to identify IBPI, avoid iatrogenic worsening during reduction and immobilisation, detect associated vascular injury, and facilitate urgent specialist referral when required [7,8]. Two British Orthopaedic Association Standards for Trauma (BOASt) documents provide the relevant national benchmarks throughout: Peripheral Nerve Injury and Diagnosis and Management of Arterial Injury Associated with Extremity Fractures and Dislocations [7,8].

This review focuses on the practical challenges facing emergency clinicians in the recognition, assessment, imaging, management, and escalation of suspected IBPI.

## Review

Methods

This article was designed as a narrative review with expert-opinion synthesis, addressing the practical challenges of recognising and managing IBPI in the emergency setting. A structured literature search of PubMed/MEDLINE was performed from database inception to February 2026, supplemented by review of reference lists and relevant national guidance documents. Search terms included combinations of *brachial plexus injury, infraclavicular brachial plexus injury, shoulder dislocation, glenohumeral dislocation, proximal humerus fracture, fracture-dislocation, axillary nerve injury, peripheral nerve injury, MR neurography, electromyography, nerve conduction studies, peripheral nerve surgery, *and *surgical reconstruction.* Priority was given to systematic reviews, clinical guidelines, cohort studies, and seminal publications relevant to the emergency recognition and management of IBPI, including literature addressing associated neurovascular complications where relevant. Consistent with the narrative review design, no formal quality appraisal or risk-of-bias assessment was undertaken. Evidence was synthesised narratively and integrated with the senior author's subspecialist expertise in brachial plexus and peripheral nerve injury to develop the proposed clinical pathway, which represents an author-derived synthesis of the available evidence rather than a formal consensus guideline.

Definition, anatomy, and injury patterns

**Figure 1:**
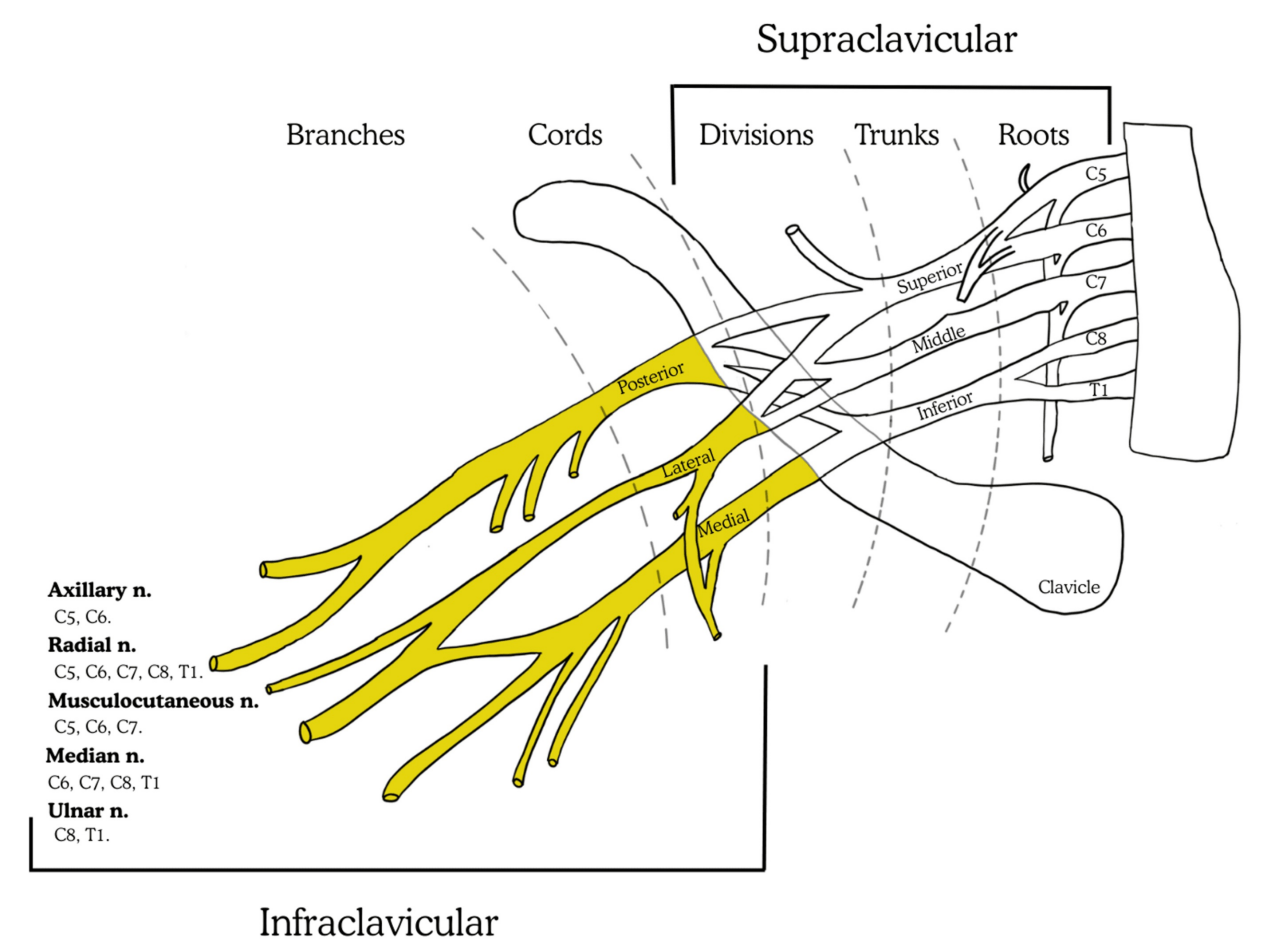
Simplified anatomy of the brachial plexus. A simplified schematic demonstrating the supraclavicular and infraclavicular regions of the brachial plexus. Created by the authors using Adobe Fresco (Adobe Inc., San Jose, California) and Canva (Canva Pty Ltd., Sydney, Australia), without the use of AI. Data from reference [10].

The brachial plexus is formed from the ventral rami of C5-T1 nerve roots, which organise into trunks, divisions, and then the lateral, posterior, and medial cords, named by their relationship to the axillary artery [9,10]. These cords give rise to the major terminal branches: the musculocutaneous, axillary, radial, median, and ulnar nerves [9,10]. Infraclavicular injuries affect the cords or their terminal branches and are anatomically distinct from supraclavicular root or trunk injuries. A simplified overview of the relevant anatomy is shown in Figure 1.

After shoulder dislocation, the axillary nerve is the most commonly affected terminal nerve in isolation [5]. Multi-nerve involvement is therefore clinically significant, raising suspicion of more extensive cord-level traction or compression rather than an isolated mononeuropathy [5,6,11].

Peripheral nerve injury severity is commonly described using the classifications proposed by Seddon and Sunderland. Seddon described three injury types: neuropraxia, a conduction block without axonal disruption that generally carries a favourable prognosis; axonotmesis, axonal disruption with an intact connective tissue framework; and neurotmesis, complete disruption of the nerve and its surrounding connective tissues [12]. Sunderland's grading system (I-V) further classifies peripheral nerve injuries according to the extent of structural disruption, including involvement of the endoneurium, perineurium, and epineurium [13]. In practical terms, neuropraxia typically recovers spontaneously, and low-grade axonotmesis may recover without surgical intervention, whereas higher-grade injuries may require reconstruction within a defined time window.

Clinical presentation

IBPI may present immediately following injury or become more apparent after analgesia or reduction, when pain-related guarding resolves and a more reliable neurological examination can be performed [11]. Patients typically report sensory disturbance (including numbness or paraesthesia), motor weakness, neuropathic pain, or loss of distal limb function. The distribution and severity of symptoms vary according to the level and extent of plexus involvement [6,9].

Motor strength should be graded using the Medical Research Council (MRC) scale, which ranges from 0 (no contraction) to 5 (normal power against full resistance) [14]. Dense motor deficits (MRC grade 0 or 1) and progressive neurological deterioration on serial examination carry a less favourable prognosis and should prompt urgent specialist assessment [9,14].

In addition to neurological findings, clinicians should actively assess for signs of associated vascular injury. These include pain out of proportion to the apparent injury, expanding axillary swelling, pallor or a cool limb, diminished or absent pulses, and a new or progressive neurological deficit in the context of suspected arterial compromise [3,8]. These features are highlighted in BOASt arterial injury standards and warrant urgent investigation and escalation because of the risk of limb-threatening ischaemia [8].

Recognising these clinical patterns and red flags requires a structured approach to assessment, which is described in the following section.

Assessment in the emergency department 

Principles and Timing

In the emergency department, priority should be given to a structured, reproducible, and well-documented assessment. IBPIs may evolve over time, and a single examination is therefore insufficient. The BOASt Peripheral Nerve Injury standards emphasise prompt initial assessment, repeated examination following any limb intervention, and clear documentation of any limitations to examination [7].

At a minimum, neurovascular assessment should be performed at four key time points: initial presentation or as soon as the patient can be adequately assessed; immediately before any intervention such as reduction; immediately after reduction or immobilisation; and once the effects of analgesia, sedation, or regional block have subsided [7,14]. Each examination should be time-stamped and recorded in a directly comparable format so that pre-existing deficits can be distinguished from iatrogenic changes and evolving pathology [7,14].

Minimum Neurological Screen

A neurological screen should be clearly mapped to the cords and terminal nerves of the brachial plexus [5,11]. Table 1 provides a structured checklist and documentation template covering sensation and motor function for each of the major terminal nerves. The same examination should be used at each assessment time point to allow meaningful comparison of findings. Where examination is limited by pain, sedation, or mechanical restriction, this should be explicitly documented together with the reason for the limitation. Any deterioration or new deficit should prompt immediate senior review and consideration of specialist referral [7].

**Table 1:** Suggested neurological assessment of the terminal branches of the brachial plexus. Sensory and motor checklist for the terminal branches of the brachial plexus. Motor power is graded on the Medical Research Council (MRC) scale (0-5). Created by the authors using Microsoft Excel (Microsoft Corporation, Redmond, Washington). Data from references [10,14].

Nerve	Sensation	Motor (with MRC grades 0-5)
Axillary	Lateral deltoid (regimental-badge area)	Deltoid contraction/shoulder abduction
Musculocutaneous	Lateral forearm	Elbow flexion (biceps)
Radial	Dorsal first web space	Wrist and finger extension ("thumbs up")
Median	Palmar surface of the index finger	Thumb opposition/adduction ("OK sign")
Ulnar	Little finger	Finger abduction (e.g., holding paper between fingers)

Concomitant Vascular Assessment

Because vascular injury can mimic, compound, or be the cause of nerve injury, a brief vascular check should be performed alongside every neurological examination. This includes capillary refill time, limb temperature and colour, and palpation of radial and ulnar pulses [8]. Where pulses are difficult to assess or equivocal, a hand-held Doppler should be used to confirm arterial flow.

Significant vascular injury may be present despite preserved distal pulses because of collateral circulation. Suspicion should therefore remain high in the presence of an associated neurological deficit, swelling, an expanding haematoma, or a high-risk fracture pattern [3,4,15-22]. Where vascular compromise is confirmed or suspected, associated nerve injury should be presumed until proven otherwise, and the vascular escalation pathway described under vascular red flags requiring urgent escalation should be followed [8]. The vascular check should be repeated after every intervention [8].

Documentation Standards

All examinations, including any limitations and escalations, should be time-stamped and clearly documented for safe clinical care and medicolegal purposes, allowing pre-existing deficits to be distinguished from iatrogenic changes and enabling deterioration to be detected promptly [7,14].

Imaging

Imaging modalities can be grouped by urgency and primary purpose. Immediate-priority investigations (plain radiographs, Doppler, CT angiography) are required to confirm diagnosis, guide safe reduction, and exclude limb-threatening arterial injury. Early investigations (CT for bony detail) define fracture morphology and guide surgical planning. Deferred investigations (MRI/MR neurography, EMG/NCS (electromyography/nerve conduction studies)) support nerve injury localisation and operative planning in the subacute phase. Table 2 summarises the recommended timing, indications, and clinical role of each modality.

Plain Radiographs

Plain radiographs are the first-line imaging modality used to confirm shoulder dislocation and its direction [23]. At least two views should be obtained, although the specific projections may vary according to patient factors such as pain and restricted movement. Once reduction has been achieved, repeat radiographs should be obtained in two planes to confirm concentric relocation and to identify associated lesions, such as a glenoid rim fracture [14,22-24].

CT Angiography

Where vascular red flags are identified, CT angiography is the imaging modality of choice and should be performed without delay [8]. BOASt arterial injury standards support integrating CT angiography into major trauma CT workflows and performing it immediately after the scout images, without repositioning the patient [8]. The presence of distal pulses does not exclude significant arterial injury; a low threshold for CT angiography is therefore appropriate when the clinical picture is concerning, particularly in fracture-dislocation or expanding axillary swelling [3,4,21,22].

CT for Bony Detail

Where radiographs suggest a glenoid rim fracture, proximal humerus fracture, or fracture-dislocation, or where clinical suspicion persists despite initial imaging, CT should be obtained to better define bony injury and guide management. Where reduction is clinically urgent, CT should not routinely delay management; however, caution is required in fracture-dislocations, where additional imaging may influence the safety of reduction and subsequent management [14,23,24].

MRI and MR Neurography

MRI is primarily used to localise nerve injury and to support operative planning. As interest in earlier surgical decision-making has increased, MRI has assumed greater importance because of its ability to identify structural nerve pathology earlier in the course of injury than electromyography, which is generally not reliable until approximately three weeks after injury [6,25]. Within emergency department care, MRI or MR neurography is most relevant when there is suspicion of a compressive lesion such as haematoma, structural entrapment in fracture-dislocation, or evolving deficits for which early surgical planning may be required [6,25-27]. MRI may also be used when there is concern for associated rotator cuff injury [24,28].

Ultrasound

Ultrasound offers a rapid, relatively low-cost method for assessing shoulder trauma and is increasingly used to assess dislocation and confirm reduction [9,26]. In brachial plexus pathology, it may identify nerve swelling or discontinuity within accessible extraforaminal segments [6,27]. However, its utility in the emergency department is limited by operator dependence and reduced visualisation of deeper structures, and it is best regarded as an adjunct rather than a replacement for MRI [6,25-27,29].

Recommended imaging modalities, timing, indications, and decision outputs are summarised in Table 2.

**Table 2:** Recommended imaging modalities, timing, indications, and decision outputs. Imaging for suspected infraclavicular brachial plexus injury after shoulder trauma is listed in order of urgency. AP, anteroposterior; CT, computed tomography; CTA, CT angiography; ED, emergency department; EMG, electromyography; MR, magnetic resonance; MRI, magnetic resonance imaging. Created by the authors using Microsoft Excel (Microsoft Corporation, Redmond, Washington).

Modality	Typical timing	Indications	Key outputs for decision-making	References
Shoulder X-ray (AP, Y, and axillary or Velpeau views)	Immediate	First-episode dislocation; suspected fracture; pre- and post-reduction confirmation	Confirms dislocation and reduction; identifies fractures and whether ED reduction is safe or CT required.	[14,23,24,30]
Doppler vascular assessment	Immediate	Equivocal pulses; cool limb; swelling; reassessment after realignment	Supports triage for CTA or vascular input.	[3,8]
CT angiography (upper limb)	Immediate (time-critical)	Any suspicion of axillary or brachial arterial injury; evolving vascular signs; combined neurovascular deficit	Detects occlusion, thrombosis, or extravasation; informs urgent vascular and orthopaedic action.	[3,8]
CT shoulder/proximal humerus	Early	Fracture suspected on X-ray; complex fracture-dislocation; pre-operative planning	Defines fracture morphology and displacement; identifies entrapment risk.	[14,23,24]
MRI brachial plexus +/- MR neurography	Deferred (typically inpatient or urgent outpatient)	Severe multi-nerve deficits; suspected compression or haematoma; surgical planning	Localises lesion; identifies structural pathology; supports early operative planning.	[6,9,23,26–28]
EMG/nerve conduction studies	Deferred (usually ≥3 weeks after injury)	Persistent deficit without clear recovery; prognostication and localisation	Defines severity and reinnervation; guides surgery versus continued observation.	[6,9,14]

Differential diagnosis

Accurate interpretation of neurological findings after shoulder trauma requires consideration of several diagnoses that can mimic or coexist with IBPI. Isolated axillary neuropraxia is the most common neurological injury following glenohumeral dislocation, accounting for 35% of nerve lesions in systematic review data [5,6]. Because the mechanism of injury alone is often insufficient to distinguish it from a more proximal infraclavicular lesion, careful clinical examination is essential. On examination, isolated axillary neuropraxia typically causes weakness of shoulder abduction and sensory changes over the lateral upper arm, whereas plexus-level injury is more likely to produce abnormalities affecting more than one terminal nerve distribution [5,6,11].

Cervical spine injury and more proximal brachial plexus lesions involving the roots or trunks should also be considered, particularly after higher-energy trauma. These injuries are anatomically and clinically distinct from infraclavicular lesions and may require a different diagnostic pathway [5,9]. Supraclavicular lesions are overall more common in traumatic brachial plexus injury, and Horner's syndrome should raise suspicion of root avulsion [9]. Where such injuries are suspected, cervical spine assessment and imaging should be undertaken in accordance with local trauma protocols, and brachial plexus MRI may need to be supplemented by cervical spine MRI to assess the intradural roots [9].

Reduced motor power may reflect pain rather than a true neurological deficit, causing apparent weakness that does not represent nerve injury. Assessment may also be limited by sedation or analgesia, adding further diagnostic uncertainty. This underlines the importance of repeat examination once the effects of analgesia or sedation have worn off [7].

Pre-existing peripheral neuropathy should also be identified, as it may complicate the interpretation of post-injury sensory and motor findings. Common examples include ulnar neuropathy at the elbow and carpal tunnel syndrome. Any such condition should be clearly documented before reduction or regional anaesthesia.

Other injuries associated with shoulder dislocation, particularly rotator cuff tears and greater tuberosity fractures, may cause pain and weakness that mimic neurological deficit. In a large prospective cohort of 3633 anterior glenohumeral dislocations, 33.4% were associated with a rotator cuff tear or greater tuberosity fracture, while isolated neurological deficit accounted for 5.8% of cases [11]. Importantly, the presence of a rotator cuff tear or greater tuberosity fracture was associated with a significantly increased likelihood of neurological deficit (relative risk 1.9; 95% CI 1.7-2.1) [11]. These findings highlight that structural shoulder injuries and neurological deficits frequently coexist rather than representing mutually exclusive diagnoses.

Acute management in the emergency department

Resuscitation and Triage

Initial management should follow standard trauma principles using an ABCDE (airway, breathing, circulation, disability, exposure) approach, ensuring that life-threatening injuries are identified and managed promptly [8,31]. Shoulder injuries occur across a wide spectrum of mechanisms, from high-energy trauma in younger patients to low-energy falls in older adults, and should be assessed within the broader clinical context [1,2]. Apparent low-energy mechanisms do not exclude significant neurovascular injury; a substantial proportion of vascular injuries in proximal humerus fractures occur following falls from standing height [3,15].

Where vascular compromise is suspected, haemorrhage control should be prioritised using direct pressure or tourniquet application, while avoiding blind clamping [8]. Early senior involvement and activation of clear referral pathways to orthopaedic and vascular services should not be delayed [8]. In cases of combined deformity and absent or diminished pulses, immediate gentle realignment and splinting should be performed, followed by repeat neurovascular assessment [7,8]. These injuries are time-critical and delay in recognition or referral may significantly affect limb salvage and functional outcome [6-8,17].

Analgesia and Anaesthesia

Effective analgesia is fundamental to the management of shoulder trauma, both to improve patient comfort and to facilitate safe reduction. Several options are available, including systemic analgesia with or without procedural sedation, intra-articular lidocaine injection, and ultrasound-guided regional anaesthesia [28,32-35]. The choice should be guided by patient factors, available expertise, and local protocols. Intra-articular lidocaine may be preferred in resource-limited settings, whereas procedural sedation and regional anaesthesia are associated with shorter procedural time and higher first-attempt success rates in some studies [28,32-35].

In the context of IBPI, analgesic choice carries an important safety consideration. Regional anaesthesia, including interscalene block, can obscure evolving neurological deficits. Where a regional block is used, a full baseline neurological examination must be documented beforehand, and re-examination planned once the block has resolved [7]. This is explicitly required by BOASt Peripheral Nerve Injury standards.

Reduction and Immobilisation

Prompt reduction is indicated when a neurological deficit is present, as ongoing traction or compression may exacerbate nerve injury [7]. A variety of reduction techniques are available, including FARES (Functional Assessment and Recovery of the Elbow and Shoulder), Spaso, and Kocher methods; current evidence does not demonstrate superiority of any single technique [36-39]. In fracture-dislocation, the reduction strategy must be guided by fracture configuration and local orthopaedic input [40].

Clinicians should be familiar with more than one technique and optimise muscle relaxation before attempting reduction [28]. Gentle, controlled movements are preferred; escalating traction or leverage against resistance should be avoided, particularly in older patients or suspected fracture-dislocation [28,36-39]. If an initial attempt is unsuccessful, an alternative technique should be used for subsequent attempts [28,37].

Following reduction of an isolated dislocation, the shoulder may be immobilised in a sling for comfort; early movement within the limits of pain is generally encouraged, and there is no clear evidence that external rotation positioning is superior to neutral [23,24]. Proximal humerus fractures are managed with collar-and-cuff or sling immobilisation according to fracture pattern and local protocol, with progressive mobilisation introduced early [40-44]. In both settings, the core principle is comfort immobilisation followed by early re-evaluation rather than prolonged rigid immobilisation [23,24,41,45].

Once reduction has been completed, the neurovascular examination must be repeated in full, clearly documented, and compared directly with the pre-reduction findings [7]. A suggested emergency department treatment checklist is provided in Table 3.

**Table 3:** Suggested emergency department treatment checklist. Practical checklist for acute assessment, documentation, imaging, and escalation. ABCDE: airway, breathing, circulation, disability, exposure; CT: computed tomography; CTA: CT angiography; MRC: Medical Research Council. Created by the Authors using Microsoft Excel (Microsoft Corporation, Redmond, Washington).

Domain	Checklist items	References
Immediate actions	ABCDE; analgesia started; limb exposed; gross deformity supported; consider C-spine precautions in high-energy mechanisms. The ABCDE framework reflects structured trauma assessment as described in ATLS and equivalent UK trauma training standards.	[8,31]
Neurovascular documentation	Named-nerve sensory mapping (axillary, musculocutaneous, radial, median, ulnar); motor screen recorded with MRC grading (0–5); pulses, capillary refill, and limb temperature documented; each examination time-stamped; repeated after any intervention.	[7–8,14,24]
Reduction/immobilisation	If dislocation with nerve deficit: prompt gentle reduction; avoid force through resistance; immobilise in sling. If proximal humerus fracture: collar-and-cuff or sling appropriate to pattern; ensure no constrictive bandages.	[7,28,36–41]
Imaging	Pre-reduction X-ray when indicated; post-reduction X-ray; CT for suspected fracture-dislocation; CTA if any vascular concern or evolving symptoms.	[3,8,14,23,24]
Analgesia plan	Document whether sedation, intra-articular lidocaine, or regional block used; baseline neurological examination before any regional block; plan re-examination after block has worn off.	[7,32-35]
Escalation triggers	Any vascular compromise; expanding axillary swelling; progressive neurological deficit; multi-nerve deficit; dense motor loss; fracture-dislocation; inability to obtain or maintain reduction.	[3,4,7,8,14,15,19-22]
Discharge safety-net	Written advice to return immediately for worsening numbness or weakness, severe pain, blue/cold hand, or increasing swelling; follow-up appointment arranged with clear time frame; referral pathway documented.	[7,9,42–46]

Indications for urgent surgery and/or transfer

Urgent operative input or transfer is required when there is a vascular red flag, an open or penetrating injury with neurological deficit, or evidence of ongoing nerve compression or entrapment that may be structurally reversible.

Vascular Red Flags Requiring Urgent Escalation

Concurrent vascular injury is uncommon but critical in suspected IBPI. A pulseless limb, ischaemic limb, active bleeding, or expanding axillary haematoma should prompt immediate senior escalation, urgent CT angiography and combined orthopaedic and vascular management [8]. If the limb is also deformed, it should be gently realigned and splinted. BOASt arterial injury standards recommend early revascularisation where feasible, with a target of less than four hours from injury [8]. A low threshold for Doppler assessment and CT angiography should be maintained in proximal humerus fracture and fracture-dislocation, since distal pulses may be preserved and delayed diagnosis risks catastrophic limb ischaemia, reperfusion-related complications, and compartment syndrome [3,4,15,17,19-22].

Compartment syndrome may complicate reperfusion following delayed revascularisation. BOASt arterial injury standards require that fasciotomy is always considered and the decision to perform, or not to, must be documented by a named senior clinician [8]. This decision should involve vascular and orthopaedic input.

Open or Penetrating Injury With Neurological Deficit

Where shoulder trauma is associated with neurological deficit and an open or penetrating injury, specialist advice should be sought within 24 hours [7,14].

Ongoing Compression or Entrapment in Fracture-Dislocation

Fracture-dislocation with concern for ongoing nerve compression or entrapment, particularly in the presence of a progressive neurological deficit, should prompt urgent orthopaedic review and consideration of reduction, stabilisation, and decompression as required. This is consistent with proposed injury mechanisms and case-based evidence describing combined neurovascular injury [3,4,15,17,19-22].

Follow-up, specialist referral, and electrodiagnostics

Patients with multi-nerve deficits, dense motor loss, progressive deficits, severe neuropathic pain, fracture-dislocation, or any vascular concern should have early referral to orthopaedics and a specialist peripheral nerve service, or to a designated single-point-of-contact pathway where available [7,9,14,46].

Delayed referral is common. In a two-centre cohort, 44% of patients experienced a referral delay of more than three months; later work suggests that pre-referral electromyography may be associated with delayed specialist presentation and late surgery [47,48]. This supports a strategy of early direct referral rather than prolonged unstructured observation or pre-referral investigation.

Time Sensitivity

Electromyography and nerve conduction studies are usually most informative only after Wallerian degeneration has developed, at around three weeks, whereas MRI may be used earlier when early surgery is being considered [6,9,25]. Emergency department discharge plans should therefore provide an explicit timetable for reassessment and referral rather than open-ended follow-up [9,14].

Prognosis and Operative Timing

Most post-dislocation brachial plexus deficits are neurapraxic or axonotmesis in nature and recover spontaneously. Complete nerve disruption is uncommon in large reviews, but a substantial minority of patients have incomplete recovery and prolonged disability [6,49]. Prognosis is influenced by the extent and severity of deficit; patient age; mechanism and energy of injury; associated fracture-dislocation complexity; vascular injury; and time to structured specialist assessment [5,6,9,46,49]. Systematic review evidence suggests superior motor outcomes when operative delay is less than six months for stretch or blunt brachial plexus injuries, while still recognising the potential for spontaneous recovery [50]. A structured pathway is therefore required to identify non-recovering lesions within this timeframe [46,50].

A summary of clinical presentation patterns, priority investigations, emergency department management priorities, and likely disposition is provided in Table 4.

**Table 4:** Clinical presentation patterns and emergency department actions. Comparison of clinical signs, priority investigations, management priorities, and expected disposition. CT: computed tomography; CTA: CT angiography; ED: emergency department; EMG/NCS: electromyography/nerve conduction studies; MRI/MR: magnetic resonance imaging/magnetic resonance; BOASt: British Orthopaedic Association Standards for Trauma. Created by the authors using Microsoft Excel (Microsoft Corporation, Redmond, Washington).

Presentation pattern	Typical clinical clues	Priority investigations	ED management priorities	Disposition and follow-up	References
Isolated axillary sensory change ± mild deltoid weakness after anterior dislocation	Lateral deltoid ("regimental badge") sensory change; often pain-limited exam; pulses normal	Pre- and post-reduction X-ray as indicated; consider ultrasound for reduction confirmation where available; consider CT if fracture is suspected on plain films	Analgesia, then gentle reduction, then recheck neurovascular status; sling for comfort; clear documentation pre- and post-intervention	Discharge if stable and improving, with an explicit re-examination plan (e.g., 7-10 days) and referral if no improvement	[5-7,14,23,24,28]
Multi-nerve deficit (radial + median/ulnar) suggesting cord-level injury	Combined distal motor/sensory deficits; dense weakness; hand intrinsic weakness; may worsen after reduction	X-ray ± CT if fracture suspected; consider early MRI/MR neurography if structural lesion suspected; plan EMG/NCS at ≥ 3 weeks	Senior review; avoid repeat traumatic reduction attempts; immobilise safely; early specialist referral pathway	Admit or urgent referral/transfer depending on severity; formal nerve-service follow-up with reassessment	[6-9,14,23,25-28,46,49]
Progressive neurological deficit or new deficit post-intervention	Deterioration on serial exam; new sensory territory involved; increasing neuropathic pain	Re-imaging for fracture displacement/haematoma; consider vascular imaging if swelling or vascular change	Escalate immediately; remove constricting dressings; reassess; urgent orthopaedic and nerve-injury advice	Urgent transfer to centre with orthoplastic/peripheral nerve expertise if needed.	[3,7-9,14,15,17,19-22,46]
Vascular concern with or without a deficit	Pulse deficit, cool limb, pallor, expanding axillary swelling/haematoma; pain out of proportion	Doppler if equivocal; CTA urgently where suspicion persists	Haemorrhage control; realign and splint, then repeat vascular exam; urgent vascular and orthopaedic involvement	Time-critical operative pathway/transfer (revascularisation targets per BOASt standards).	[3,4,7-9,14,15,17,19-23,46]
Proximal humerus fracture/fracture-dislocation with neurovascular symptoms	Gross deformity; swelling; neurological deficit; pulses may be present initially, then lost	X-ray and CT for fracture pattern; CTA if any vascular concern or evolving findings	Analgesia; immobilise (collar-and-cuff or sling per pattern); avoid forceful manipulation; repeat neurovascular exams	Admit/urgent orthopaedic review; vascular involvement if CTA positive or clinical concern.	[3,8,9,14,23,24,32,33,36,39-41,45]

Proposed emergency department pathway for suspected IBPI

**Figure 2:**
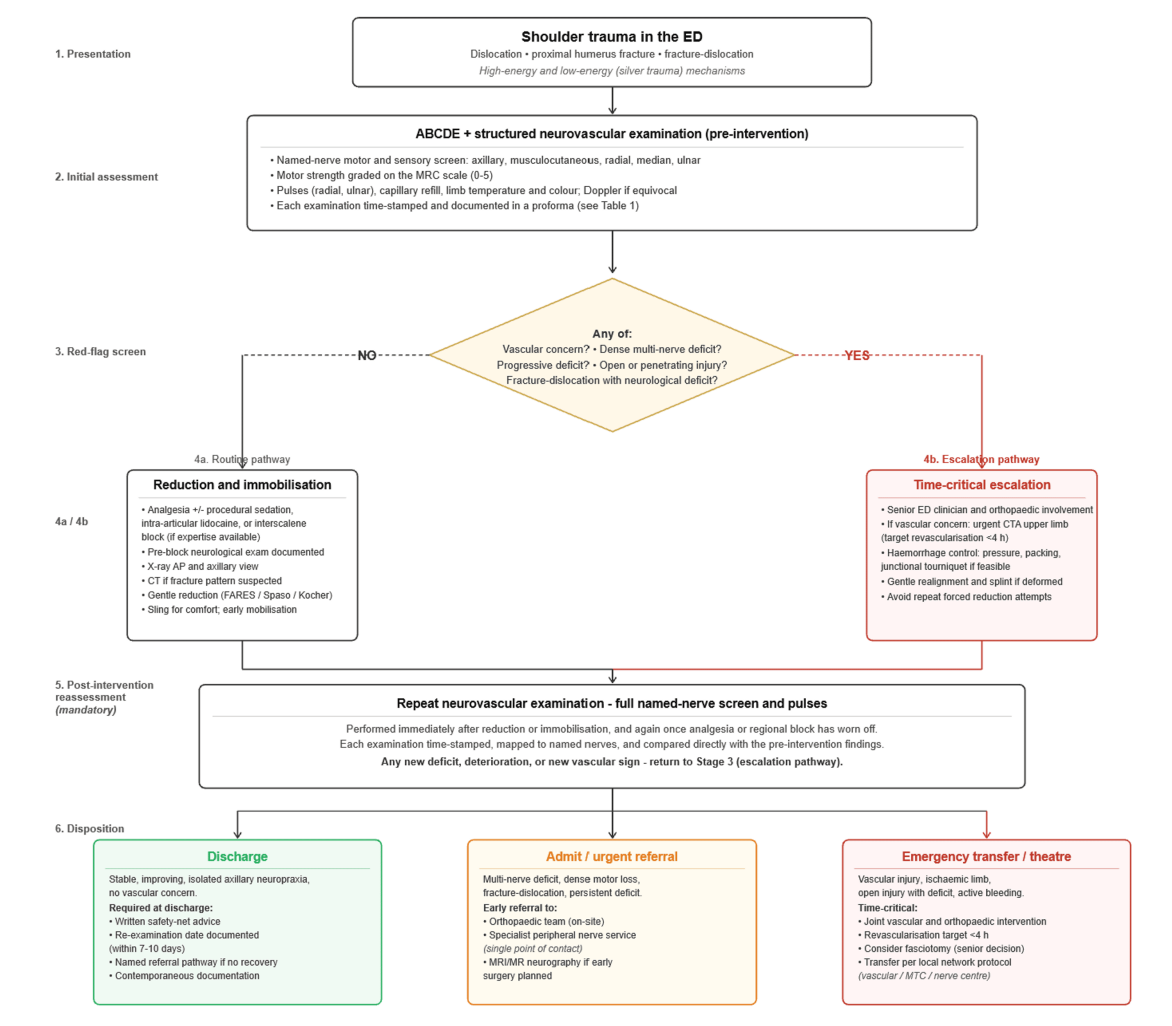
Proposed emergency department pathway for suspected infraclavicular brachial plexus injury after shoulder trauma. Pathway integrating pre- and post-intervention neurovascular examination, red-flag escalation screen, and disposition criteria. Adapted from BOASt Peripheral Nerve Injury and Arterial Injury standards [7,8]. Intended for local adaptation and audit. ABCDE: airway, breathing, circulation, disability, exposure; CTA: CT angiography; ED: emergency department; FARES: Functional Assessment and Recovery of the Elbow and Shoulder; MRC: Medical Research Council; BOASt: British Orthopaedic Association Standards for Trauma. Created by the authors using Canva (Canva Pty Ltd., Sydney, Australia), without the use of AI. Data from references [3,7-9,12-25,27,31-38,40-48,50].

A consolidated emergency department pathway for suspected IBPI is shown in Figure 2. The pathway is intended as a practical reference tool and should be read alongside the checklist in Table 3 and the clinical presentation summary in Table 4. It integrates structured pre- and post-intervention neurological examination; a red-flag screen covering vascular and neurological escalation triggers; mandatory reassessment after every intervention; and disposition decisions. The pathway is adapted from BOASt standards for Peripheral Nerve Injury and Arterial Injuries [7,8] and is intended for local adaptation and audit rather than as a validated evidence-based algorithm. As noted in the Methods, this is an expert-opinion synthesis and has not been prospectively validated.

Medicolegal and systems considerations

Missed or poorly documented peripheral nerve injuries are a recognised source of clinical negligence claims. Aggregate data on "Nerve Damage" claims published by NHS Resolution under the Freedom of Information Act, reviewed by the authors and corrected as at 31 January 2022, list Emergency Medicine among the specialities with closed claims in the period 2009/10 to 2020/21 [51].

From a systems perspective, BOASt standards emphasise clear pathways, single points of contact, and documented reassessment after interventions [7,8]. These elements can be incorporated directly into emergency department checklists and proformas.

Strength of the evidence base

Table 5 summarises the principal evidence underpinning each clinical domain of this review, with study designs, sample sizes, the headline finding, and an overall level of evidence. Most domains rest on systematic reviews and large observational studies; reduction and analgesia are supported by randomised and pooled trial evidence, whereas vascular injury and several management points rest on case-level data and expert standards. The grades are read alongside the caveat in each row and the Limitations below.

**Table 5:** Evidence base and strength of supporting evidence, by clinical domain. Studies are identified by first author and year; full citations appear in the reference list. CI: confidence interval; ED: emergency department; EMG: electromyography; LoE: level of evidence; OR: odds ratio; RCT: randomised controlled trial; FARES: Functional Assessment and Recovery of the Elbow and Shoulder. Levels of evidence (adapted from OCEBM): 1, systematic review or meta-analysis of randomised trials; 2, individual randomised trial or prospective cohort, or systematic review of cohorts; 3, retrospective cohort or case-control; 4, case series or review of case-level data; 5, narrative review or expert opinion. A range indicates that the domain rests on studies spanning those levels. Table created by the authors using Microsoft Excel (Microsoft Corporation, Redmond, Washington).

Clinical domain	Principal evidence (design; n)	Headline finding	LoE	Strength of evidence and main caveat
Epidemiology of shoulder dislocation and proximal humerus fracture	Chen 2024 and Liu 2026 (secondary analyses of Global Burden of Disease data); Shah 2017 (UK cohort, 16,763); Leroux 2014 (cohort, 20,719); Liavaag 2011 (incidence study)	Crude incidence about 75 per 100,000 (2019), bimodal with peaks in young men and older women; proximal humerus fractures mainly affect older adults	2-3	Consistent across large population studies; provides context only. Ecological and coding-based estimates
Nerve injury after glenohumeral dislocation (frequency, pattern, prognosis)	Lorente 2023 (systematic review, 17,087); Robinson 2012 (prospective cohort, 3,633); Hardie 2023 (retrospective cohort, 243); Gutkowska 2018 and 2020	Deficit in about 6 to 13.5% (isolated deficit 5.8%); axillary nerve commonest; multi-nerve involvement signals cord-level injury; most recover, a minority incompletely	2-3	Moderate. Reported rates vary widely (heterogeneous examination and electrodiagnostic practice); retrospective predominance
Vascular injury with proximal humerus fracture or fracture-dislocation	Menendez 2015 (population-based, US); Shepherd 2024 (systematic review of 55 cases); Pena 2024 (prospective, 31 of 105); case reports and series	Rare (0.09 to 5%) but limb-threatening; may occur with palpable pulses; strongly associated with brachial plexus injury (OR 109); complex fractures higher risk	2-4	Low to moderate. Best single estimate is population-based; most detail is case-level and prone to publication bias
Imaging (MRI, ultrasound, electrodiagnostics)	Chin 2018 (diagnostic systematic review, 7 studies); Szaro 2022, Griffith 2019, Pacella 2025 (radiology reviews)	MRI reliable for traumatic plexus (sensitivity 93%, specificity 72%); ultrasound a useful adjunct (less reliable for C8 and T1); EMG unreliable before about 3 weeks	2-5	Moderate for MRI; lower for ultrasound (operator-dependent). Few diagnostic studies; remainder narrative
Reduction technique	Gonai 2023 (systematic review with network meta-analysis, 14 RCTs and 1,189 patients); Sayegh 2009 (RCT, 173); Lee 2025 (RCT, 32); Gottlieb 2020 (review)	No single manoeuvre is universally superior; gentle, low-force techniques (for example FARES) compare favourably for success, pain, and time	1-2	High that no method dominates; technique choice is pragmatic. Individual trials small
Analgesia and anaesthesia for reduction	Fitch 2008 (systematic review of intra-articular lidocaine versus sedation); Zitek 2022 (RCT, 43); Blaivas 2011 (RCT, 42); Raeyat Doost 2017 (RCT, 60)	Intra-articular lidocaine and procedural sedation give similar reduction success, with possibly fewer adverse events from lidocaine; ultrasound-guided interscalene block may shorten length of stay	1-2	Individual emergency department trials are small and single-centre; a more recent meta-analysis would strengthen this domain
Referral and surgical timing	Martin 2019 (systematic review of individual patient data, 569); Zhang 2021 (cohort, 84); Yang 2025 (cohort, 99); Hill 2021, Bateman 2025, Morris 2022 (reviews and commentary)	Best motor recovery with surgery within 6 (ideally 3) months; delayed referral common (44%); pre-referral EMG associated with later presentation and surgery	2-3	Moderate. Pooled and single-centre retrospective data with referral bias; consistent direction of effect
Immobilisation	Tunnicliffe 2024 (systematic review, 39 studies and 2,664 patients); BESS/BOA and ED guidance	No clear advantage of any sling type or duration; comfort immobilisation with early movement is preferred	2-3	Moderate. Heterogeneous studies; evidence is for conservatively managed fractures

Limitations

Evidence for ED-specific IBPI pathways remains limited by heterogeneous retrospective studies; variable definitions of nerve injury; inconsistent reporting of neurological examination, imaging, electrodiagnostics, and outcomes; and mixed injury phenotypes [3,5]. This is acknowledged in systematic reviews of nerve injury after dislocation and vascular injury after proximal humerus fracture [3,5]. The predominance of retrospective observational designs throughout the underlying evidence base means that selection bias and referral bias are plausible confounders, and that incidence and prognosis estimates vary substantially across studies. Neurological assessment methods are inconsistent across the cited literature, with some studies relying on clinical examination alone and others incorporating electrodiagnostic testing, limiting direct comparability. No formal risk-of-bias assessment was performed, meaning that the quality of individual studies contributing to recommendations has not been systematically graded. The proposed pathway has not been prospectively validated for feasibility, reliability, or impact on patient outcomes, and external validation in a prospective multicentre design would be required before formal implementation as a clinical guideline.

## Conclusions

The most useful single step in improving emergency department recognition of IBPI is a structured, time-stamped neurological examination, mapped to named cords and terminal branches and performed before and after every intervention. This is the core principle of the UK BOASt Peripheral Nerve Injury standards. It makes deterioration detectable, protects the window for reconstructive surgery, and produces a defensible clinical record.

IBPI after shoulder trauma is uncommon, but the consequences of missed or delayed recognition can be severe and may include persistent weakness, sensory loss, neuropathic pain, and functional impairment. Alongside structured serial examination, the wider ED priorities are prompt but gentle reduction when a nerve deficit is present, analgesia that does not obscure baseline deficits, recognition of associated vascular red flags requiring urgent escalation, and early referral to a specialist peripheral nerve service rather than open-ended outpatient follow-up. The pathway and checklists proposed here provide a starting point for local adaptation and audit, and prospective evaluation is needed.
